# Glass hybrid versus resin-modified glass ionomer cement in class II restorations of primary molars: a 24 months randomised clinical trial

**DOI:** 10.1007/s40368-026-01184-0

**Published:** 2026-02-16

**Authors:** P. Papadopoulou, V. Siokis, I. Kotsanos, V. Boka, A. Arhakis, N. Kotsanos

**Affiliations:** Aristotle University of Thessaloniki, Thessaloniki, Greece

**Keywords:** Paediatric dentistry, Primary molar, Class II restoration, Glass ionomer cement

## Abstract

**Purpose:**

To compare the 24 month outcomes of a newer version of conventional glass ionomer cements (GIC; Equia Forte; GC Corp., Tokyo, Japan) and a standard resin-modified glass ionomer cement (RMGIC; Fuji II LC; GC Corp.) in Class II primary molar restorations and investigate reasons for failures.

**Methods:**

Healthy and cooperative 4- to 9-year-old children with proximal surface caries lesions in all four primary molars of the same jaw, following clinical and/or radiographic examination, received Class II restorations with GIC or RMGIC, allocated randomly per quadrant. These were assessed semi-annually for 2 years using modified USPHS criteria. The radiographic examination was conducted annually, and assessed the presence of furcation/periapical radiolucencies, pathological root resorption and secondary caries lesion formation. Cox regression analyses were used to determine restoration survival between the two materials; Mc Nemar’s test was used to compare proportions of failure between the two restorative materials. The level of statistical significance was set at *p* < 0.05.

**Results:**

Of the 63 children initially included in the study, 55 attended all four recalls, with a total of 120 restorations per material. There was a statistically significantly higher success rate in the RMGIC group compared to GIC (90.8% and 66.7%, respectively, *p* < 0.001). Most failures of GIC were attributed to partial or total loss of restoration (31.7%).

**Conclusion:**

RMGIC Class II primary molar restorations had statistically significantly greater success compared to GIC restorations in the 24 month observation period.

## Introduction

Glass ionomer cements (GICs) were first introduced as dental materials during the 1970s (Wilson and Kent 1972). Originally, they were the result of an acid (phosphoric)—base (glass) reaction that formed a setting paste with many disadvantages, including low resistance to fracture, high early wear, increased sensitivity to moisture, long setting times and also aesthetic considerations (Burke et al. 2023). Since then, efforts were made to improve the clinical characteristics of GICs, whilst maintaining their main advantages, such as biocompatibility, similar thermal expansion as the tooth, adhesion to enamel and dentine, and fluoride release (Rizzante et al. 2015; Dias et al. 2018; Mustafa et al. 2020).

During the 1990s, material modifications such as high viscosity GIC and resin-modified glass ionomer cements (RMGICs) were introduced, having improved physical characteristics, for more predictable prognosis compared to their predecessors. Nowadays, RMGICs are materials that combine advantages from both GICs and resin composites; some materials have triple setting characteristics that harden by 1) the initial light curing, 2) chemical curing, and 3) the longer neutralisation reaction (maturation) (Berg and Croll 2015). RMGICs have advanced physical properties compared to conventional GICs and perform well in typical Class II primary molar restorations for 2–3 years (Chadwick and Evans 2007, Hubel and Mejare 2003, AAPD 2023). The most common causes of failure for RMGICs in Class II restorations in children are reported to be partial loss of retention and secondary caries lesion formation (Chadwick and Evans 2007; Santos et al. 2010; Dermata et al. 2018).

In an effort to improve the mechanical properties of conventional GICs without the addition of resin, thus rendering the material independent of the need for light polymerisation, the high viscosity GICs were introduced. Having smaller glass particles, higher powder/liquid ratio, quicker setting, lower sensitivity to moisture, increased hardness, and allegedly higher fracture resistance, these materials have been reported to be a viable alternative to resin composite for Class I and small Class II restorations in permanent teeth for up to 3 years (Cribari et al. 2023). However, there is limited evidence regarding the success of contemporary, referred to as ‘highly viscous’ or ‘glass hybrid’, GICs in Class II primary molar restorations.

Class II cavities in primary molars face specific challenges, including restoration fracture risks by placing often relatively large restorations in smaller teeth, caries risk not sufficiently controlled due to challenges with young children’s oral hygiene and diet, and cavity preparation and material placement difficulties in the less compliant child patient. For those reasons, the need for biocompatible, quick-setting materials with low sensitivity to humidity, acceptable wear and fracture resistance that are relatively easy to place and aesthetically appealing is intensified when a tooth coloured option is desired.

Systematic reviews highlight the need for well-designed randomised controlled trials (RCTs) in order to increase evidence for the use of contemporary high viscosity GICs in primary molar restorations (Chisini et al. 2018; Dias et al. 2018; Siokis et al. 2021). Equia Forte (GC Corp., Tokyo, Japan) was first introduced in 2015 as an improved version of the Equia (Fuji IX GP Extra & G-Coat plus; GC Corp.) system. According to the manufacturers, this newer ‘glass hybrid’ version had smaller and more reactive glass fillers, and increased flexural strength, surface hardness and wear resistance (Equia Forte GC brochure www.gcasia.info).

The aim of the present split-mouth RCT was to assess the clinical performance of a glass hybrid GIC (Equia Forte), compared to a RMGIC (Fuji II LC; GC Corp.), in Class II primary molar restorations for 24 months. Thus, the primary outcome of the study was to compare restoration success between Fuji II LC and Equia Forte. The null hypothesis was that there is no statistically significant difference between the two materials. The secondary outcome was to investigate which parameters affected failures within the observation period.

## Materials and methods

### Ethical aspects

This study was approved by the Dental faculty ethics committee of Aristotle University of Thessaloniki (32/31-3-2016). Prior to their enrolment in the study, all participants and their legal guardians were informed about the possible risks and benefits. Informed written consent was signed by every legal guardian.

### Sample size and materials

To calculate the appropriate sample size, a power analysis was performed with the program G*Power version 3.1. The sample size was defined based on the desired statistical power (80%) and an acceptable error level of *α* = 0.05, while a moderate effect size (0.3) was chosen. The necessary sample size, as calculated by the power analysis, was 144 teeth, i.e. 72 per material group. Another 20% was added to account for potential losses; therefore, 44 was the minimum number of children to be enrolled in the study. In order to account for loss of patients from recruitment to completion of the operative phase, and any possible attrition within the required 24 month follow-up period, possibly from early tooth exfoliation etc., it was decided to enrol all eligible children attending the university paediatric dentistry postgraduate program clinic during the second and third semester of the program and continue until all remaining follow-up appointments were completed. A total of 63 patients were therefore initially enrolled in the study. Details of the materials used in the study are given in Table 1.

**Table 1:** Materials used in the study

Material	Type	Content	Producing company
GC Equia Forte Fil	Glass hybrid cement	Powder: 95% strontium fluoro alumino-silicate glass, 5% polyacrylic acid Liquid: 40% aqueous polyacrylic acid	GC Industrial Corp. (Tokyo, Japan)
Equia Forte Coat	Multifunctional monomer	40%–50% methyl methacrylate, 10%–15% colloidal silica, 0.09% camphorquinone, 30%–40% urethane methacrylate, 1%–5% phosphoric ester monomer	GC Industrial Corp. (Tokyo, Japan)
GC Fuji II LC	Resin modified glass ionomer cement	Powder: 100% fluoro-alumino-silicate Liquid: 35% HEMA, 25% distilled water, 24% polyacrylic acid, 6% tartaric acid, 0.1% camphoroquinone, BisGMA and traces of TEGMA	GC Industrial Corp. (Tokyo, Japan)

### Study design, blinding and randomisation

In order to avoid bias related to the difference in caries risk at the proximal areas (Cagetti et al. 2011), it was decided that any molar proximal carious surface to be restored would contact a carious surface of the other primary molar, diagnosed clinically or radiographically. This was feasible because it is a very frequent phenomenon for high caries risk children to have carious lesions affecting both the distal of the first as well as the mesial surface of the adjacent second primary molar.

The present study was a two-arm split-mouth randomised controlled trial. Healthy and cooperative 4 to 9 year-old children attending Aristotle University paediatric dental department, with occluso-proximal caries lesions in all four primary molars of the same jaw following clinical and/or radiographic examination, received treatment with Equia Forte or Fuji II LC. Random assignment was done using the calculator at https://www.randomizer.org/. Each quadrant was assigned a unique number, and the tool generated a random sequence of numbers to ensure an equal and random distribution of teeth into the two groups. The treatment was performed by any of six postgraduate students of the department under the supervision of three experienced faculty members. This was a triple-blinded RCT, as all trial participants, outcome assessors and data analysts were blinded to the interventions; the operators could not be so, due to capsule identification of differences in the materials.

### Eligibility criteria

Following approval of this protocol, all patients attending the department were assessed for eligibility to enrol. This was done with the standard clinical and radiographic examination. The inclusion criteria were: healthy and cooperative children (ASA I, II) with no systematic disease or syndrome and without special health care needs. Children were free from any developmental tooth disorder, especially hypomineralised second primary molars (HSPM) as this could affect the adhesion of the materials, the risk of caries lesion development, and the restoration survival (Kotsanos et al. 2005; Voinot and Bedez 2024). Each participant had two adjacent primary molars (maxillary or mandibular) with Class II carious lesions requiring restoration in at least two dental quadrants, as explained previously in the study design. Radiographically, the dentinal carious lesion could reach the inner half of dentine but not contacting the pulp. The teeth should be asymptomatic or diagnosed as having reversible pulpitis (no or only provoked symptoms from the pulp, together with absence of swelling, abscess and mobility). A tooth preparation, following caries tissue removal, that included central areas of cusps or exceeded buccal and lingual proximal angles constituted a criterion for exclusion. Another exclusion criterion was patients and/or legal guardians not committed to attend follow-ups.

### Clinical procedures

Following informed consent, eligible children were treated; each by the assigned postgraduate student. Topical anaesthetic EMLA 5% (Aspen Pharma, Ireland) was applied for a few minutes and local anaesthesia using 2% lidocaine with 1:80,000 adrenaline (Lignospan, Septodont, France) was administered. Cavity preparation was performed using a sterile No. 330 high-speed bur and water irrigation. The carious lesion was then removed using a No. 4 or 5 sterile round steel bur in a slow-speed handpiece, depending on cavity size, without water irrigation. Following the removal of soft dentine, there was only partial removal of harder affected carious dentine to minimise risks of pulp exposure during cavity preparation (Kotsanos and Arizos 2011). A custom cut 4 mm wide metal matrix band and a small wooden wedge were placed and the cavity was dried but not desiccated. The two teeth from the same quadrant were restored randomly with either GIC or RMGIC according to the manufacturer’s instructions. Excess material was removed during handling time and a resin coat placed and light cured at 800 mW cm^−2^ for 10 s in the case of Equia Forte. The matrix was carefully removed sideways only after full material setting 3 min later, as was done with the RMGIC, which was similarly light cured for 20 s. The restorations were checked by the experienced supervisors if they could be rated A by every USPHS criterion (Table 2), otherwise the restoration was redone. This was also required by the Program standards. In the following appointment within two weeks, the opposite quadrant of the same jaw was restored using the same procedures with the alternative material. All stages of treatment, from cavity preparation to final restoration, were performed under rubber dam isolation according to standard practices in University specialty programs (Varughese et al. 2016).

**Table 2:** Modified USPHS criteria used in this study

Category	Score	Criteria	
	Acceptable	Unacceptable	
Retention of restoration	A		Presence
		C	Total or partial absence
Marginal adaptation	A		Absence of crevice along the periphery of the restoration
	B		Not visible crevice along the periphery of the restoration
		C	Visible crevice along the periphery of the restoration
Marginal discoloration	A		Absence
		C	Presence
Secondary caries	A		Absence
		C	Presence
Gingival health	A		Absence of bleeding
	B		Bleeding on probing
		C	Automatic bleeding
Abrasion of occlusal surface	A		Absence of abrasion
	B		Abrasion without tooth cut-surface exposure
		C	Abrasion with tooth cut-surface exposure

### Clinical and radiographic evaluation

The evaluation of the treatment was done in a blind manner by the main investigator. The evaluation of the restorations was based on the clinical criteria of the United States Public Health Service (USPHS), modified for the needs of the study (Table 2).

Restorations were clinically rated as A = Alpha (excellent), B = Bravo (acceptable), C = Charlie (unacceptable). Criteria were modified slightly for the needs of the study. For example, the criterion for contact surface was purposely deleted as in other similar studies on primary teeth (Kotsanos and Arizos 2011), because primary teeth often migrate or exfoliate during a study. Radiographic criteria (in addition to clinical criteria) were: absence of furcation/periapical lesion, including widening of the periodontal ligament space and loss of lamina dura, absence of internal/external pathological root resorption and absence of secondary caries lesions.

Clinical evaluations were performed at 6, 12, 18, and 24 months, whilst radiographic examinations were undertaken at 12 and 24 months. Restorations that received scores A or B at 24 months were considered successful. Restorations with a clinical score C in at least one criterion, except for marginal discoloration, were considered unsuccessful (failed), as were those with clinical and/or radiographic evidence of pathology and were censored at the time of failure in the survival analysis calculation.

### Data statistical analysis

The McNemar’s test for paired data was used to compare the proportions of failure between the two restoration materials. Kaplan–Meier curves and cox regression models with robust standard errors clustered at the individual (patient) level to account for multiple measurements for each patient were used to determine restoration survival between the two materials. Bonferroni correction was used to adjust for multiple comparisons. The level of significance was set at *p* < 0.05. The annual failure rates (AFR) for RMGIC and for GIC restorations were estimated based on the methodology of Opdam et al. (2014).

## Results

**Fig. 1:**
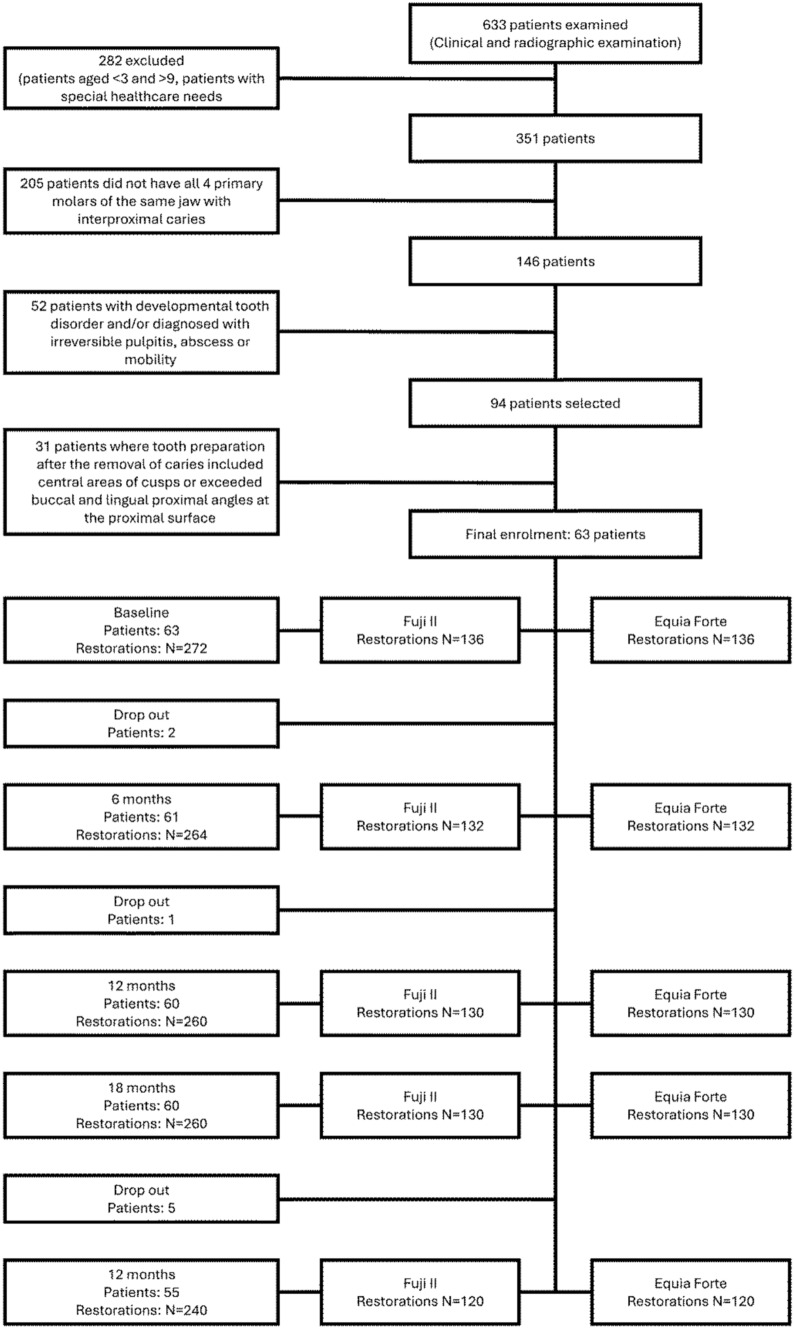
Consort diagram of the clinical study

**Fig. 2:**
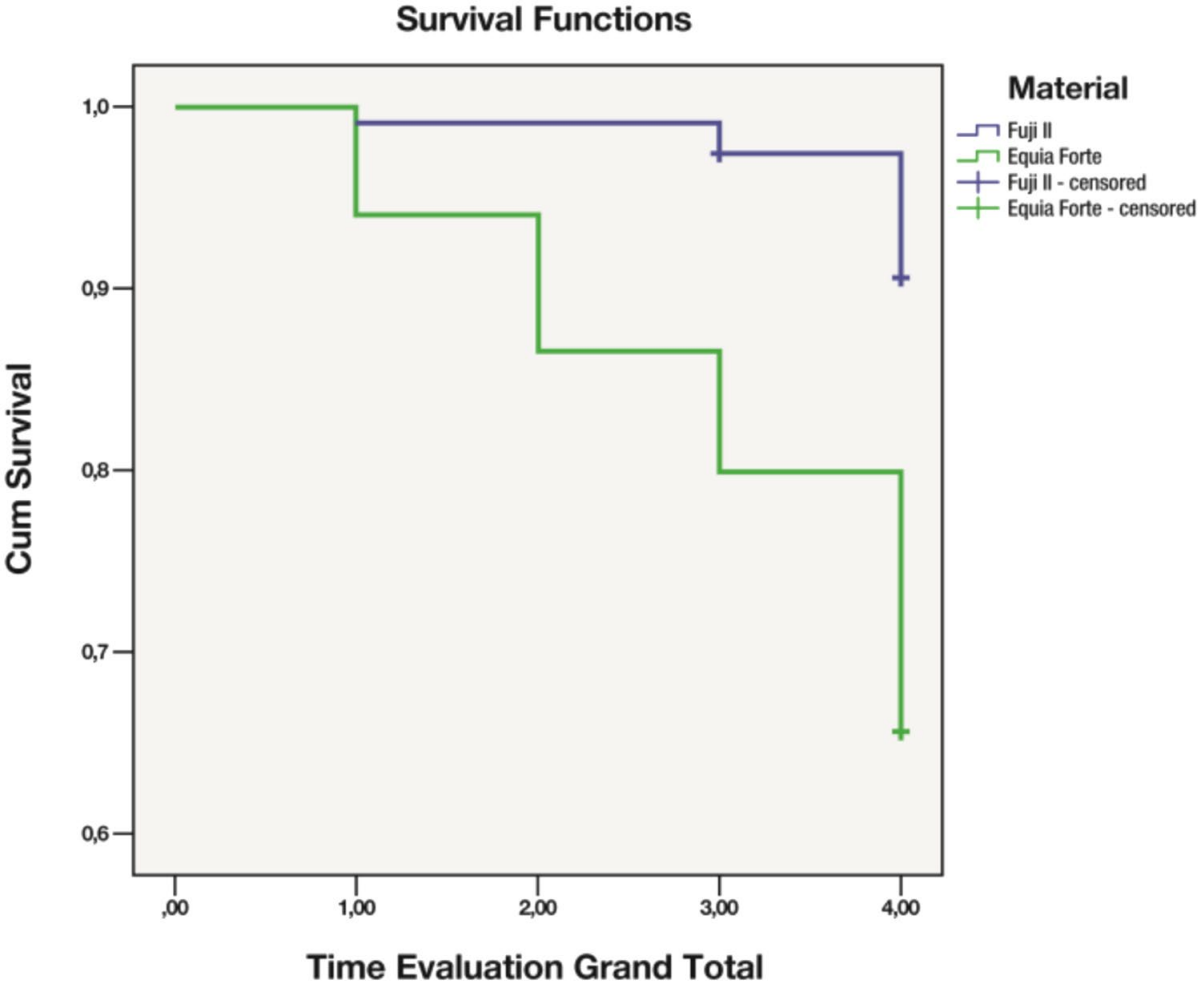
Kaplan–Meier analysis of restoration survival at the four follow-ups

A total of 272 restorations were placed initially in 63 children, 36 boys and 27 girls, with a mean age of 6.51 (SD = 1.34). The restorations were performed in the period between March 2016 and February 2017 and the last follow-up was completed in February 2019. Eight participants had dropped out by the end of the study; two after baseline, one after the 1st and five after 3rd follow-up. Reasons for not attending their follow-ups were changing work circumstances and having to move away. As a result, 240 restorations in 55 children were evaluated throughout the 2 year recall period (Fig. 1). In five children, restorations were performed in all primary molars.The number of restorations according to tooth type and material placed are decribed in Table 3. The overall success rate for the 24-month follow-up period was higher (90.8%) in RMGIC restorations compared to the 66.7% for GIC restorations (*p* < 0.001). The cumulative survival per material for the total duration of this study can be found in Fig. 2. Restorations with RMGIC material had better survival than restorations with GIC material (restoration survival probability (95% CI) for RMGIC vs. GIC: 0.92 (0.88, 0.97) vs 0.65 (0.58, 0.73), *p* < 0.001). The annual failure rate for RMGIC restorations was estimated at 4.7%, whereas for GIC it was 18.3%.

**Table 3:** Number of restorations according to tooth type and material placed

		RMGIC	GIC	TOTAL PER JAW
1st Primary molars	Maxillary	31 (50.0%)	31 (50.0%)	62 (100%)
	Mandibular	29 (50.0%)	29 (50.0%)	58 (100%)
2nd Primary molars	Maxillary	31 (50.0%)	31 (50.0%)	62 (100%)
	Mandibular	29 (50.0%)	29 (50.0%)	58 (100%)

The frequency of distribution and percentage of reasons for failure for each material, based on the clinical criteria, are presented in Table 4. In 64.8% of cases, failure of restoration for both materials was due to loss of retention. Additionally, two GIC restoration failures were diagnosed through radiographic examination; one had furcation pathology at 12 months, while the other had secondary caries lesion formation at the final examination. First primary molars had a significantly higher failure rate than second primary molars (28.3% vs. 14.2%, *p* = 0.011). Particularly, maxillary first primary molars had increased failure compared to the other teeth (32.8% vs. 21.3%, *p* = 0.012).

**Table 4:** Reasons for failure per material over 24 months

Clinical Evaluation (Modified USPHS Criterion)	RMGIC		GIC		*P* value
	Number*	%	Number*	%	
Retention (C)	10	8.3	38	31.6	< 0.001
Marginal Adaptation (C)	1	0.8	9	5.8	0.011
Marginal Discoloration (C)	0	0	3	3.5	0.083
Secondary Caries (C)	1	0.8	7	7.5	0.021
Gingival Health (C)	0	0	0	0	-
Abrasion (C)	1	0.8	0	0	0.317

*Restorations could have failure in more than on clinical evaluation/ Modified USPHS Criteria, but in total sum only one failure was taken into account

In terms of retention, as a clinical criterion for success, for the total of 24 months follow-up period, the RMGIC restorations had a success rate of 91.7%, whereas the GIC restorations had a success rate of 68.4% (*p* < 0.001). Failure due to loss of retention differed statistically significantly at all recalls except for the first one (Table 5). Kaplan–Meier survival analysis showed that retention of restorations was similar for maxillary and mandibular primary molars. Cox regression analysis showed that the restorations with GIC had 4.5 times greater risk of failure due to loss of retention at 24 months compared to RMGIC restorations (HR = 4.59, 95%CI 2.36, 8.94, *p* < 0.001). The tooth type also affected the survival of a restoration regarding this criterion (Table 6). When partial versus total loss of retention was examined, the difference was not statistically significant between the two studied materials (*p* = 0.729).

**Table 5:** Restoration failure due to loss of retention through all four 6-monthly clinical evaluations

Recall	Group	Unsuccessful *N* (%)	*p* value
6 months	FUJI II LC	1 (0.8)	0.063
	EQUIA FORTE	6 (5.0)	
12 months	FUJI II LC	0 (0.0)	0.003
	EQUIA FORTE	9 (8.0)	
18 months	FUJI II LC	2 (1.7)	0.125
	EQUIA FORTE	7 (6.9)	
24 months	FUJI II LC	7 (6.1)	0.007
	EQUIA FORTE	16 (16.8)

**Table 6:** Univariate Cox regression analysis for restoration failure due to loss of retention

Characteristic	HR	95% CI	*p* value
Age	0.93	0.65, 1.32	0.6
Material			
FUJI II LC	Ref		
EQUIA FORTE	4.59	2.36, 8.94	< 0.001*
Mandible			
Maxillary	Ref		
Mandibular	0.67	0.32, 1.44	0.31
Molar			
First	Ref		
Second	0.44	0.28, 0.69	< 0.001*
Side			
Right	Ref		
Left	0.73	0.39, 1.37	0.33

HR = Hazard Ratio, CI = Confidence Interval

^*^Statistically significant at level 5%

There was a statistically significant difference in the marginal adaptation between RMGIC and GIC at every review. The failed GIC restorations for this criterion at 24 months were 9.2% compared to 0.9% for RMGIC (*p* = 0.005). Eleven GIC and one RMGIC restorations had a visible marginal gap with exposed dentine, in which case they were replaced. In 10 of those cases, the marginal gap was accompanied by a secondary caries lesion (as defined by the discoloured exposed dentine, data not tabulated).

Marginal discoloration was similar at the 6 and 12 month evaluation between the two materials (Pearson chi-square); however, three GIC restorations had increased marginal discoloration on the 3rd follow-up examination compared to RMGIC (*p* = 0.05). None of the RMGIC restorations presented discoloured margins during the 24 month period. Regarding gingival health, there was no statistically significant difference between the two materials for the 24-month study duration. Six teeth had bleeding on probing at the last two examinations and were scored as B = Bravo; two with RMGIC and four with GIC restorations.

**Fig.3:**
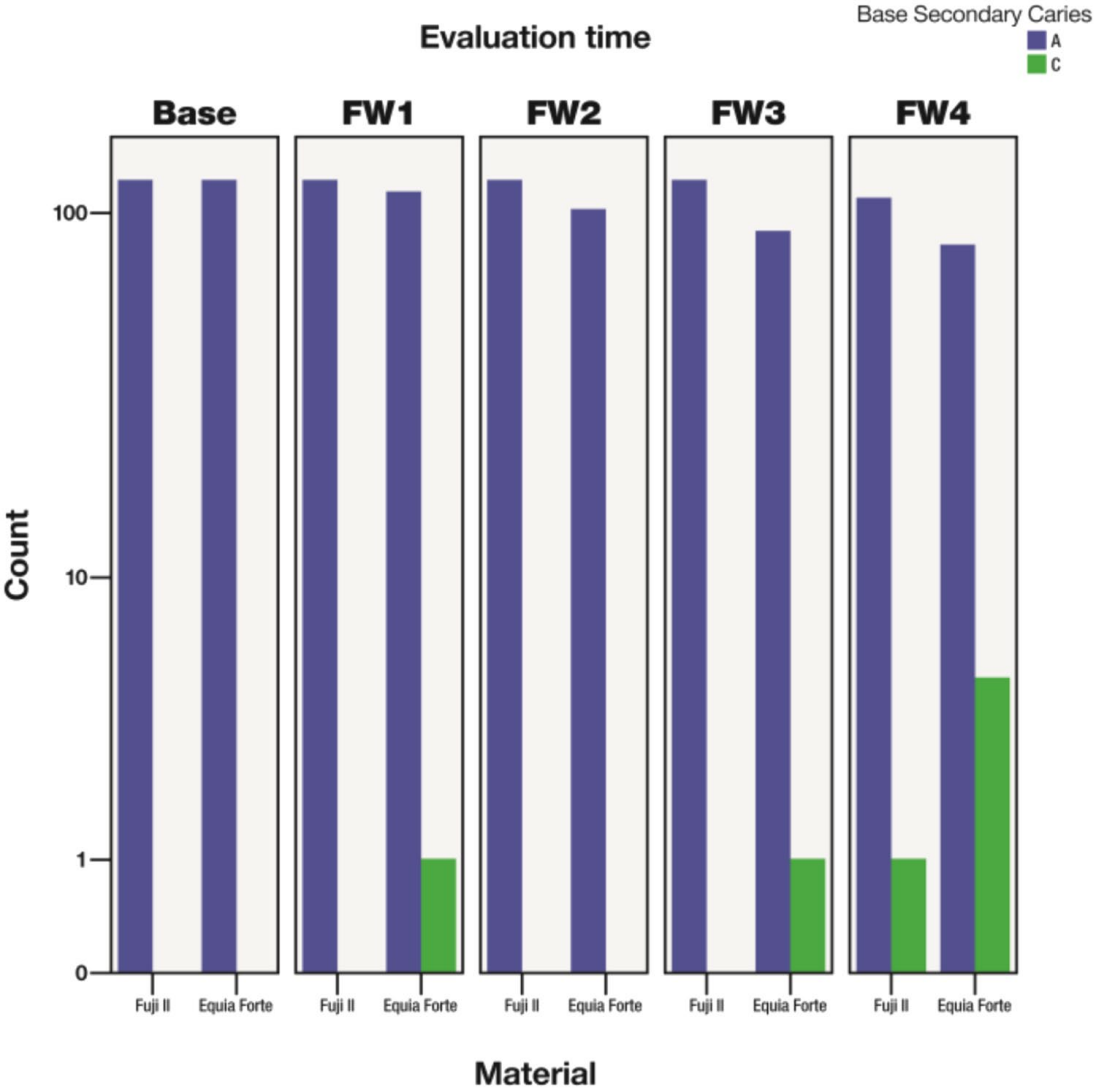
Secondary caries lesion present in restorations with RMGIC and GIC at each follow-up

Throughout the whole study period, one RMGIC restoration had secondary caries lesion formation, whilst nine GIC restorations (9.6%) failed due to caries lesion development. For most teeth this was at the final recall (Fig. 3) and the difference between the materials was statistically significant (*p* = 0.045) at the final recall. For GIC restorations, the secondary caries events differed statistically by the end of 24 months compared to baseline, 1st and 2nd follow-ups in the within-group analysis.

**Fig.4:**
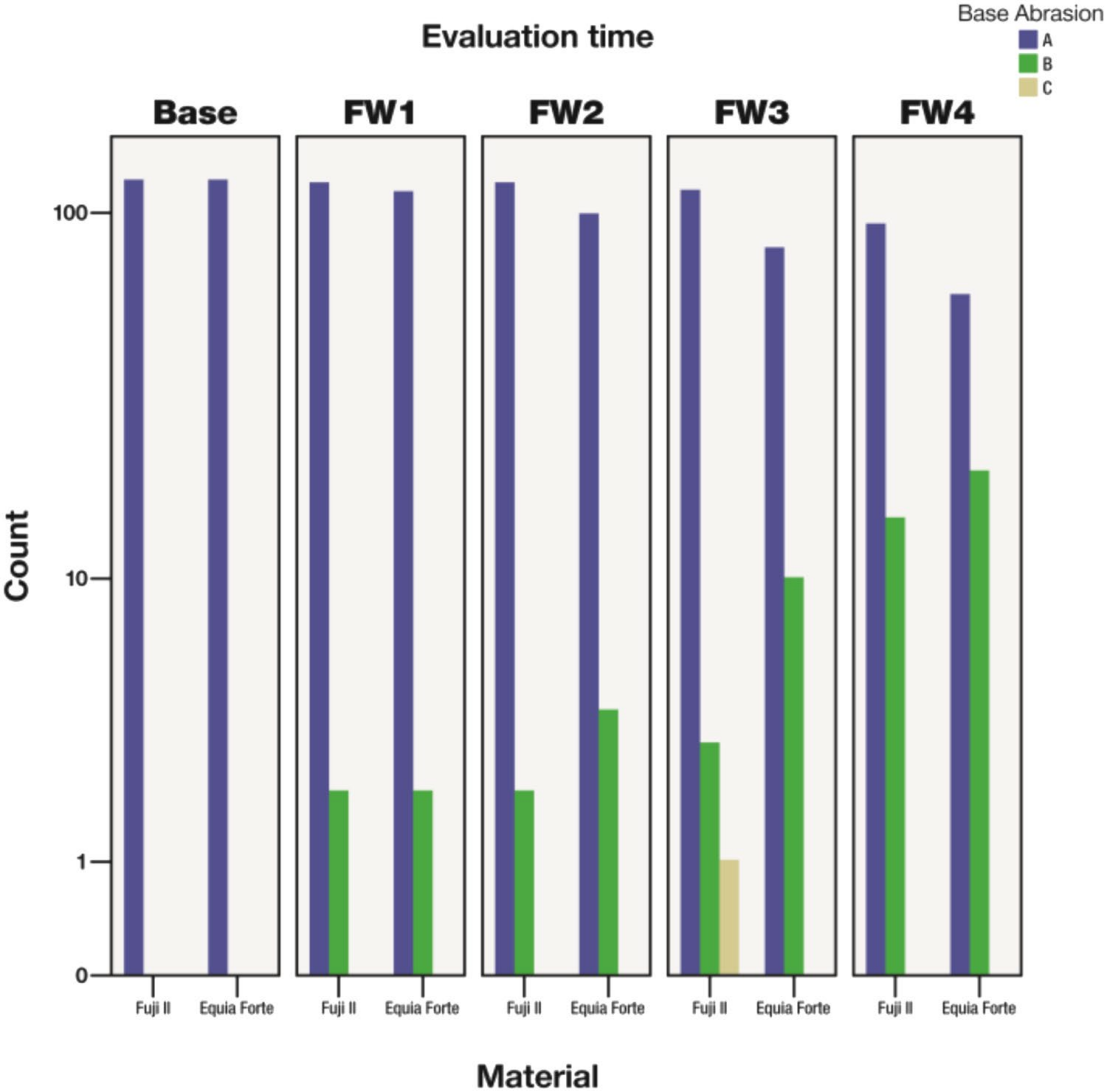
Bar chart showing the scores of abrasion for each type of material tested, at each follow-up examination

The difference in abrasion between the two materials was found to be statistically significant (*p* = 0.03) at the 18 month follow-up evaluation; however, this was not the case at the final assessment, as the wear of RMGIC restorations had quintupled from the 18 month evaluation (Fig. 4). One GIC restoration showed abrasion leading to dentine exposure and was replaced at the 18 month follow-up (Fig. 4). In all other cases, restoration abrasion did not expose any dentine and the scores were B = Bravo.

Cox regression analysis showed that the restorations with GIC had 4.7 times greater risk to fail due to any of the examined criteria, compared to RMGIC restorations (HR = 4.71, 95%CI 2.42, 9.17, *p* < 0.001) The tooth type was also found to affect the survival of a restoration due to any of the criteria. More specifically, tooth 55 had lower risk for failure by 71% than tooth 54 (HR = 0.29, 95%CI 0.11, 0.75, *p* = 0.01); tooth 74 had lower risk for failure by 67% than tooth 54 (HR = 0.33, 95%CI 0.10, 1.05, *p* = 0.060); tooth 75 had lower risk for failure by 78% than tooth 54 (HR = 0.22, 95%CI 0.06, 0.79, *p* = 0.020) and tooth 85 had lower risk for failure by 77% than tooth 54 (HR = 0.23, 95%CI 0.06, 0.88, *p* = 0.032). Finally, second primary molars had lower risk for failure by 57% than first primary molars (HR = 0.43, 95%CI 0.27, 0.68, < 0.001) (Table 7).

**Table 7:** Univariate cox regression analysis for total failure

Characteristic	HR	95% CI	*p*-value
Age	0.96	0.71, 1.30	0.7
Material			
FUJI II LC	Ref		
EQUIA FORTE	4.71	2.42, 9.17	< 0.001*
Tooth type			
54	Ref		
55	0.29	0.11, 0.75	0.010*
64	0.60	0.25, 1.45	0.26
65	0.39	0.14, 1.11	0.078
74	0.33	0.10, 1.05	0.060
75	0.22	0.06, 0.79	0.020*
84	0.84	0.33, 2.14	0.71
85	0.23	0.06, 0.88	0.032*
Mandible			
Maxillary	Ref		
Mandibular	0.71	0.34, 1.49	0.36
Molar			
First	Ref		
Second	0.43	0.27, 0.68	< 0.001*
Side			
Right	Ref		
Left	0.71	0.38, 1.31	0.27

HR = Hazard Ratio, CI = Confidence Interval

*Statistically significant at level 5%

## Discussion

The null hypothesis of the present study was rejected, as there was a statistically significant difference in the survival rate between the two materials. The results regarding GIC are similar to those of previous studies that investigated the system Equia Fil and Coat (GC Corp., Japan, Tokyo), the predecessor of this material (Radu et al. 2019; Akman and Tosun 2020). Radu et al. (2019) reported an annual failure rate of 13.3% for this GIC, while Akman and Tosun (2020) reported 82% success of Equia restorations in 12 months, similar to the present 87.5% for the GIC material for the same period.

A 2018 international meeting in Bauru, Brazil, reached a consensus regarding conventional GIC thresholds for restorative indications (de Lima Navarro et al. 2021). For the parameters evaluated in vitro, Equia Forte was ranked first in mechanical properties, amongst 18 different GIC materials; it was, however, found to be more vulnerable in acidic erosion. The more acidic environment in proximal areas, the difficulty to apply the coat evenly, and the possible damage to the surface integrity of the material while removing the matrix band after placement of restoration (Scholtanus et al. 2007, Kupietzky et al. 2019), could partially explain the survival rates of the GIC restorations in the present study. Manufacturers recommend Vaseline application on the matrices prior to their placement, in order to avoid their adherence to the material (GC technical brochure).

Further to material characteristics, there are other factors which can also affect restoration success in paediatric dentistry, including cavity type, size and form, previous intervention for the tooth, conditioning of the cavity prior to restoration, complete or incomplete caries tissue removal, patient compliance, caries risk, and last but not least, ability and experience of the operator (Dias et al. 2018; Chisini et al. 2018; Amend et al. 2022). In the authors’ opinion, if a more consolidated form of cavity avoiding any hint of occlusal isthmus was preferred (frequently called ‘box only’), GIC restorations might suffer less from fracture and partial loss of material.

All children in the present study had high caries risk, and individuals with higher caries risk have shorter survival times for Class II direct restorations (Laske et al. 2016; Chisini et al. 2018). This was controlled for by the split-mouth design in a uniform sample of high caries risk patients. However, it could be a study limitation, as the results may not be representative for use in low to medium caries risk children. All treatments were carried out by six postgraduate students with the same training and similar experience; however, the results may have differed if a more experienced paediatric dentist had placed the restorations, which could be another limitation of this study.

To the authors’ knowledge, this is the first split-mouth RCT evaluating this glass-hybrid GIC in Class II restorations in primary molars over 2 years. Previous long-term studies of GIC found high survival rates for small-medium Class II restorations in permanent posterior teeth (Wafaie et al. 2022). Gurgan and colleagues studied GIC in an adult cohort and reported that more than 90% of restorations in extended Class II cavity preparations were successful for at least 24 months (Gurgan et al. 2020). The positive results in permanent teeth can be attributed to the size of the cavities, which are large enough for bulkier restorations, which may be less prone to fracture (Berg and Croll 2015).

Most of the failures in the present study related to loss of retention were found in first maxillary primary molar teeth, in agreement with previous studies (Ghaderi et al. 2015, Baba et al. 2021). This could be because of less remaining sound hard dental tissue thickness in the proximal areas of this tooth, which may compromise the retention of the restoration. Such reasons call for alternative restorative philosophy when addressing large and/or deep cavities in these teeth and this is their full coverage with preformed metal crowns. To satisfy aesthetics, there are also zirconia crowns but these are more invasive in tooth preparation as well as demanding regarding chairside time and cost.

Baba et al. (2021) reported that 74.4% of Class II primary molar restorations with GIC survived the studied period of 12 months; the retention loss was higher compared to the present 12 month data. One reason might be the rubber dam isolation used in the present study. Literature has contradictory results regarding the type of isolation and its role on the success of a restoration; a Cochrane review from 2021 reported that rubber dam attributes positively to the survival of the restoration (Miao et al. 2021), whereas other reviews did not find any difference in the success of a restoration when rubber dam or cotton roll isolation were used (Dias et al. 2018; Chisini et al. 2018).

Another study by Erdogan and Sonmez (2021) found only 9.4% of success for GIC Class II restorations in primary molars at 12 months; however, this study did not use the GIC Coat, but an alternative resin-free coat. In vitro studies have shown the critical role of GIC Coat in improving the material surface hardness and limiting progressive wear as well as discoloration (Poornima et al. 2019; Mohan et al. 2021).

In the present study, the annual failure rate of RMGIC was lower than the median annual failure rate for RMGICs in primary molar restorations in a recent systematic review, where Amend et al. (2022) reported that the annual failure rate for RMGICs in restorations of posterior primary teeth ranged between 1.9 and 16.9%. For high viscosity GICs, the rate ranged between 2.9 and 25.6%. The present results on GIC fall in this spectrum, with an annual failure rate of 18.7%. A meta-analysis of in vitro evaluation of adhesion of GICs to primary tooth enamel and dentine found that RMGICs bond more strongly than conventional and high viscosity GICs (Peric et al. 2021), which could be a putative reason for the increased success rate of RMGIC compared to GIC restorations in the present study.

The present results are not transferable to the newest GIC HT materials and this may be a limitation for the present study. This, however, may be inherent to other similar studies and owes to the frequent replacement/evolution of restoration materials by manufacturers, taking into account the years necessary for designing, performing and following up such clinical trials. A very recent study of the latter material has promising results for its use in permanent teeth restorations for 18 months, as it appeared to reach the minimum 90% success threshold rate indicated by ADA (Atmaca and Karadas 2024). However, to the authors’ knowledge, there is no published RCT regarding the use of GIC HT in restorations of primary molar teeth. Therefore, clinical evaluation of this newer material is warranted in the field of paediatric dentistry.

## Conclusions

- RMGIC Class II primary molar restorations had statistically significant greater success than GIC restorations in the 24 month observation period.
- The most prevalent cause of failures was partial/total loss of restoration retention.
- Restorations in maxillary first primary molars experienced higher failure rates regardless of the material placed.

## Data Availability

No datasets were generated or analysed during the current study.
